# Supplementary material for: Mapping a 50-spin-qubit network through correlated sensing
Source: Nat Commun. 2024 Mar 5;15:2006. doi: 10.1038/s41467-024-46075-4 (PMC10914733; doi:10.1038/s41467-024-46075-4)
Supplement: Supplementary file 1 — Supplementary Information [file 41467_2024_46075_MOESM1_ESM.pdf]

# Supplementary Information for "Mapping a 50-spin-qubit network through correlated sensing"

**Supplementary Table I**: Information on the nuclear spins mapped in this work. A dash denotes that no $\Delta_i$ signal could be obtained due to low polarisation, coherence, or readout contrast. Value in parentheses denotes standard deviation on the last digit. Spins C1-C27 were previously characterised in Ref. [1] and the used labels are consistent with their work. These data are also available online at: *https://doi.org/10.4121/aba1cc84-0aea-4cdc-93ca-68b0db38bd81.v1*

| Label | Initialisation | Readout | $A_i$ (kHz) | $\Delta_i$ (Hz) | x (Å) | y (Å) | z (Å) |
|---|---|---|---|---|---|---|---|
| C1 | PulsePol | Chain-1 | 452.83(2) | -20840.2(6) | 0.0 | 0.0 | 0.0 |
| C2 | PulsePol | Chain-1 | 455.37(2) | -22939.6(1) | 2.52 | 2.91 | -0.51 |
| C3 | PulsePol | Chain-1 | 463.27(2) | -31257.8(1) | 3.78 | 0.73 | -0.51 |
| C4 | PulsePol | Chain-1 | 446.23(4) | -14056.7(2) | -1.26 | 2.18 | 0.0 |
| C5 | SWAP | Direct | 447.234(1) | -11291(3) | 0.0 | 4.37 | -6.18 |
| C6 | SWAP | Direct | 480.625(1) | -48488.0(8) | 5.04 | -1.46 | -2.06 |
| C7 | PulsePol | Chain-1 | 440.288(6) | -8332.8(1) | 5.04 | -1.46 | 5.66 |
| C8 | PulsePol | Chain-1 | 441.77(1) | -9803.6(8) | 7.57 | 1.46 | 3.6 |
| C9 | SWAP | Direct | 218.828(1) | 213147.2(2) | 7.57 | -4.37 | -10.81 |
| C10 | SWAP | Direct | 414.407(1) | 17643.3(4) | 0.0 | 8.74 | -12.36 |
| C11 | SWAP | Direct | 417.523(4) | 14549.91(4) | 6.31 | 9.46 | -12.87 |
| C12 | SWAP | Direct | 413.477(1) | 20546.7(3) | 11.35 | 0.73 | -14.42 |
| C13 | PulsePol | Chain-1 | 424.449(1) | 8017.1(2) | 12.61 | 2.91 | -6.69 |
| C14 | SWAP | Direct | 451.802(1) | -19760.5(3) | 5.04 | -2.91 | -22.65 |
| C15 | PulsePol | Chain-1 | 446.01(5) | -13958.0(3) | 1.26 | 3.64 | -22.65 |
| C16 | PulsePol | Chain-1 | 436.67(5) | -4647.8(1) | 2.52 | 8.74 | -23.17 |
| C17 | PulsePol | Chain-1 | 437.61(1) | -5682.1(1) | 6.31 | -2.18 | -29.34 |
| C18 | SWAP | Direct | 469.02(1) | -36184.3(2) | 0.0 | -1.46 | -19.05 |
| C19 | SWAP | Direct | 408.317(1) | 24219.15(8) | 3.78 | -9.46 | -8.75 |
| C20 | PulsePol | Chain-1 | 429.403(4) | 2692.5(5) | 3.78 | 10.92 | -4.63 |
| C21 | PulsePol | Chain-1 | 430.937(3) | 1214.8(4) | -5.04 | 5.82 | -4.12 |
| C22 | PulsePol | Chain-1 | 424.289(3) | 7696.07(9) | 16.39 | -3.64 | -8.24 |
| C23 | PulsePol | Chain-1 | 435.143(7) | -3195.6(1) | 13.88 | 0.73 | 5.66 |
| C24 | PulsePol | Chain-2 | 436.183(3) | - | 1.26 | -0.73 | 9.78 |
| C25 | PulsePol | Chain-2 | 435.829(5) | - | 7.57 | 1.46 | 9.78 |
| C26 | PulsePol | Chain-1 | 435.547(2) | - | 12.61 | -5.82 | -0.51 |
| C27 | PulsePol | Chain-1 | 435.99(3) | -3935.9(2) | 1.26 | -3.64 | -31.4 |
| C28 | PulsePol | Chain-1 | 440.9(1) | -8915.47(3) | -1.26 | 2.18 | -24.71 |
| C29 | PulsePol | Chain-1 | 434.3(1) | -2185.7(1) | -6.31 | 0.72 | -19.05 |
| C30 | PulsePol | Chain-1 | 427.1(1) | 4871.11(4) | 12.62 | 10.19 | -14.93 |
| C31 | PulsePol | Chain-1 | 428.3(1) | - | 11(4) | 15(4) | -11(4) |
| C32 | PulsePol | Chain-1 | 431.6(1) | - | 6(3) | 12(2) | -3(9) |
| C33 | PulsePol | Chain-1 | 439.0(1) | - | -2.52 | -1.46 | 4.12 |
| C34 | PulsePol | Chain-1 | 437.3(1) | - | -2.52 | -0.0 | 6.18 |

Continued on next page

**Supplementary Table I**: Information on the nuclear spins mapped in this work. A dash denotes that no $\Delta_i$ signal could be obtained due to low polarisation, coherence, or readout contrast. Value in parentheses denotes standard deviation on the last digit. Spins C1-C27 were previously characterised in Ref. [1] and the used labels are consistent with their work. These data are also available online at: *https://doi.org/10.4121/aba1cc84-0aea-4cdc-93ca-68b0db38bd81.v1*

| Paper label | Initialisation | Readout | $A_i$ (kHz) | $\Delta_i$ (Hz) | x (Å) | y (Å) | z (Å) |
|---|---|---|---|---|---|---|---|
| C35 | PulsePol | Chain-1 | 427.4(1) | 4591.33(4) | 20(3) | -1(7) | -8(1) |
| C36 | PulsePol | Chain-1 | 434.4(1) | -2214.2(8) | -8(7) | -0(7) | -23(9) |
| C37 | PulsePol | Chain-1 | 429.1(1) | 2899.5(2) | 13(2) | -12(1) | -18(1) |
| C38 | PulsePol | Chain-1 | 434.0(1) | - | 12.61 | -8.73 | 0.0 |
| C39 | PulsePol | Chain-2 | 432.5(1) | -450(5) | 16(5) | -8(5) | -24(4) |
| C40 | PulsePol | Chain-2 | 433.3(1) | -1173(5) | 11(3) | -14(3) | -26(4) |
| C41 | PulsePol | Chain-2 | 434.1(1) | -2189.3(6) | 8(4) | -12(4) | 2(6) |
| C42 | PulsePol | Chain-2 | 434.8(1) | - | 10.09 | -7.28 | -28.83 |
| C43 | PulsePol | Chain-2 | 432.2(1) | -270.1(6) | 11(3) | 7(3) | -19(3) |
| C44 | PulsePol | Chain-2 | 433.9(1) | -1882.8(6) | -0.0 | 11.65 | -26.77 |
| C45 | PulsePol | Chain-3 | 436.2(1) | -4174(1) | 6.3 | 6.56 | -29.35 |
| C46 | PulsePol | Chain-3 | 434.8(1) | - | 14(4) | -5(4) | -24(5) |
| C47 | PulsePol | Chain-2 | 429.4(1) | 2587.8(3) | 21(4) | -7(7) | -14(7) |
| C48 | PulsePol | Chain-2 | 431.0(1) | - | 16(4) | -4(4) | -21(9) |
| C49 | PulsePol | Chain-2 | 428.3(1) | 3744.4(2) | 23(4) | -4(8) | -12(3) |
| C50 | PulsePol | Chain-2 | 436.2(1) | -4227.0(4) | 11(7) | 5(7) | -2(9) |

**Supplementary Table II**. Spin labels of spins featuring in the experiments in the main text.

| Figure | Spin number | Spin label |
|---|---|---|
| Fig. 2 | Spin 1 | C18 |
| | Spin 2 | C28 |
| | Spin 3 | C16 |
| | Spin 4 | C44 |
| | Spin 5 | C45 |
| Fig. 3 | Spin 1 | C11 |
| Fig. 4 | Spin 1 | C12 |
| | Spin 2 | C13 |
| | Spin 3 | C22 |

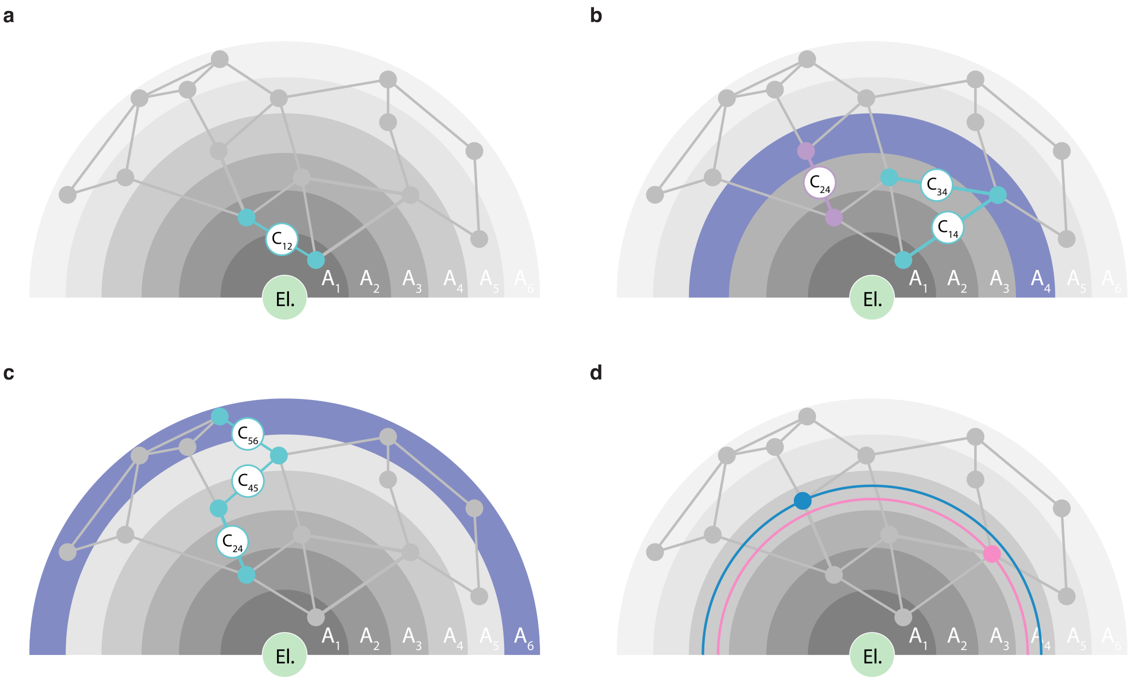


**Supplementary Fig. 1**. *Mapping complex spin networks.* a) Spatial representation of Fig. 1, specific to the electron-nuclear system. Nuclear spins (dots) are connected by lines denoting observable couplings ($C_{ij} > 1/T_2$). The bands indicate spin frequencies $A_i$, shifted by the hyperfine interaction $\Delta_i$ with the electron spin (mint green). This interaction diminishes with distance from the NV center, leading to spectral crowding (multiple spins per band). For simplicity we do not visualize the angular dependence of the hyperfine interaction. Frequencies $A_1$, $A_2$ and $A_3$ contain only a single spin, allowing for a direct readout with the electron spin. Additionally, the measured couplings between these frequencies (e.g. $C_{12}$) can be assigned unambiguously from pairwise measurements (see Fig. 1a). b) When multiple spins occupy the same frequency band (e.g. at $A_4$), spin-chain measurements resolve ambiguity by retrieving the connectivity of the network (see also Fig. 1b). c) Spins in spectrally crowded areas (e.g. at $A_6$, see also Supplementary Fig. 1), can still be accessed via a spin-chain, starting from a directly accessible spin (e.g. at $A_2$) (see Fig. 1c). d) High-resolution measurements of $\Delta_i$ (indicated by narrow pink and blue bands) allow for directly distinguishing multiple spins in a single frequency band (e.g. at $A_4$) (see Fig. 1d). Note that we use different frequency labels compared to Fig. 1.

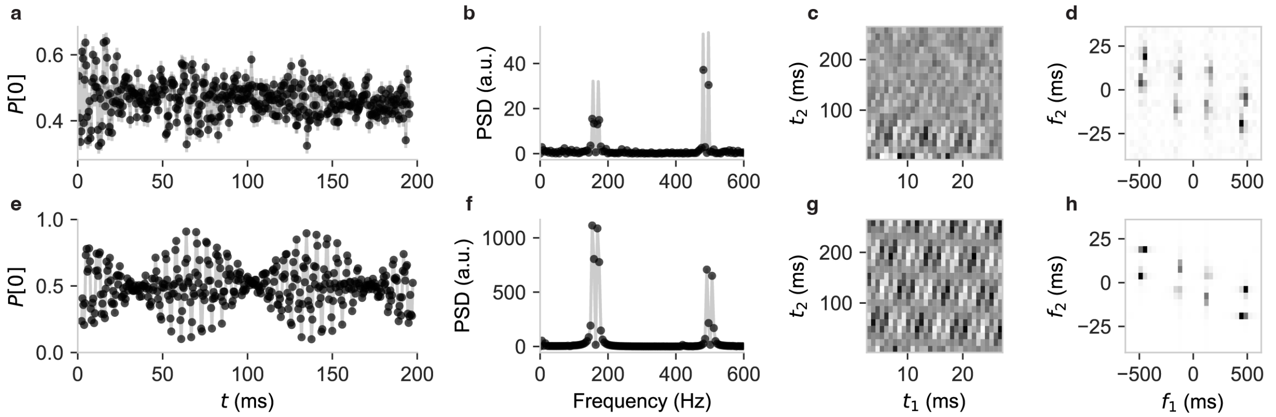


**Supplementary Fig. 2**. *Comparison to numerical simulations.* a) Time domain data of the experiment in Fig. 4d and corresponding PSD (b). c) Time domain data of the experiment in Fig. 4e and corresponding 2D PSD (d), showing both the positive and negative $f_1$-axis. e-h) Numerical simulations of the experimental data in (a-d). Spin parameters are based on the spin positions. The simulations show good agreement with experiment (up to nuclear decoherence effects), reconfirming the characterisation of the system and the interpretation of the spectroscopy data.

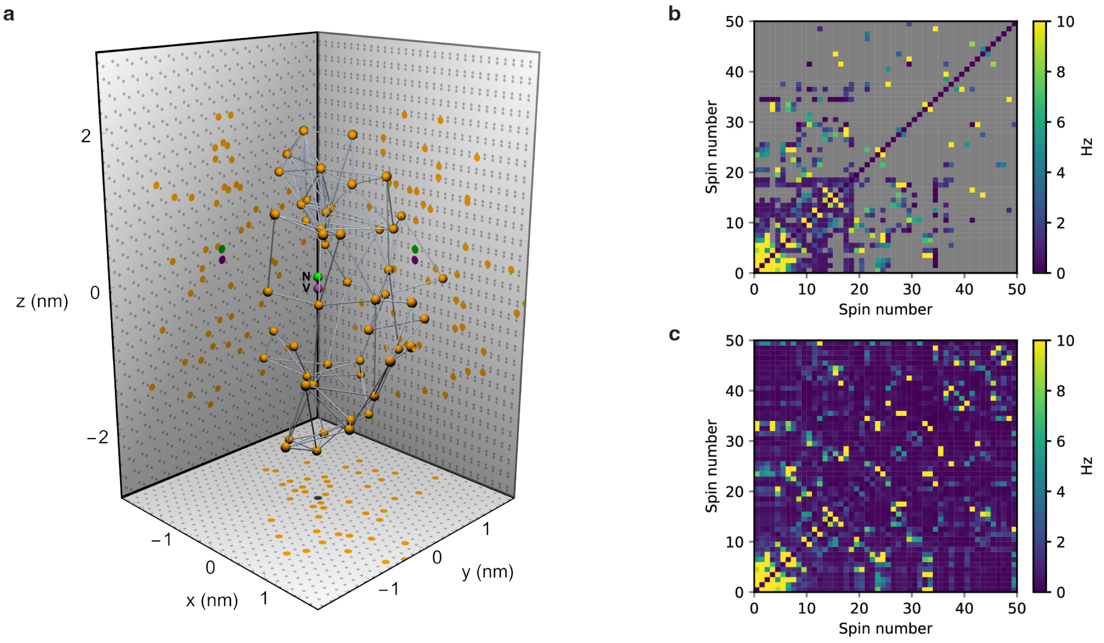


**Supplementary Fig. 3**. *Spatial structure of the 50-spin network.* a) Most likely positions for the 50 $^{13}$C nuclear spins mapped in this work. Couplings larger than 3 Hz are visualised by the grey connections. The NV vacancy site is placed at the origin. b) Measured coupling matrix. Elements that were not measured, or did not return a clear signal (due to spectral crowding) are colored grey. c) Predicted coupling matrix, based on the most likely spin positions (Methods).

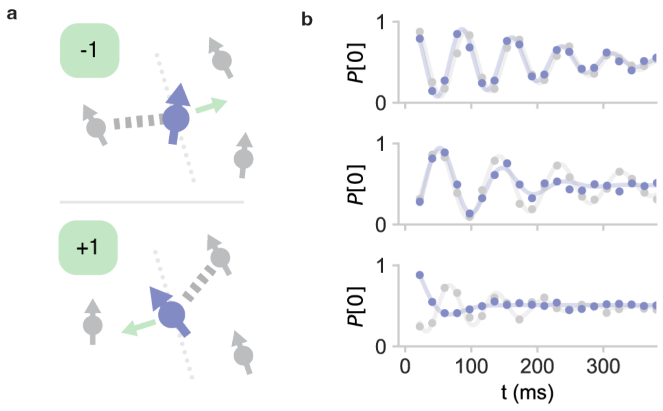


**Supplementary Fig. 4**. *Electron-state-dependent dephasing* a) Schematic of the dephasing effect for a target nuclear spin (purple), coupled to a local spin bath (grey) under the electron-nuclear double resonance sequence (see Fig. 3). Due to the perpendicular hyperfine component (mint-green arrow), the nuclear quantisation axes change when the electron changes spin state (from $|-1\rangle$ to $|+1\rangle$). As a result, the nuclear spin couples differently to its environment in the first and second half of the spin-echo, limiting the effectiveness of the echo if the environment is in an unpolarised state. b) Experimental data of the electron-nuclear double resonance experiment (as in Fig. 3) for three different nuclear spins (C19, C18 and C5). The surrounding spin-bath is either initialised in a mixed state (purple) or polarised 'up' (grey). Solid lines are fits to the data (as in Fig. 3). For some spins (most notably C5), the described dephasing effect leads to a quick drop in coherence, which can be partially regained by polarising the bath. This effect can be accurately modelled based on the extracted spin positions (see Supplementary Fig. 7). The data is corrected for the difference in global and selective polarisation direction.

## Supplementary Note 1: NV system

### 1. Hamiltonian

We consider the Hamiltonian of the ground-state NV electron spin, surrounded by $N$ $^{13}$C nuclei [2]:

$$\hat{H} = \Delta_{\mathrm{ZFS}}\hat{S}_z^{\;2} + \gamma_{\mathrm{e}} B_{\mathrm{z}} \hat{S}_z + \sum_{i=1}^{N} \gamma_{\mathrm{c}} B_{\mathrm{z}} \hat{I}_{\mathrm{z}}^{(i)} + \sum_{i=1}^{N} \hat{\vec{S}} \cdot \boldsymbol{A}^{(i)} \cdot \hat{\vec{I}}^{(i)} + \sum_{i=1}^{N}\sum_{j=i+1}^{N} \hat{\vec{I}}^{(i)} \cdot \boldsymbol{C}^{(ij)} \cdot \hat{\vec{I}}^{(j)}\,, \tag{1}$$

with $\Delta_{\mathrm{ZFS}}$ the zero-field splitting, $\gamma_{\mathrm{e}}$ and $\gamma_{\mathrm{c}}$ the electron and $^{13}$C nuclear gyromagnetic ratio and $B_{\mathrm{z}}$ an external magnetic field applied along the NV-symmetry axis (z-axis). Here, $\hat{\vec{S}} = (\hat{S}_x, \hat{S}_y, \hat{S}_z)$ and $\hat{\vec{I}}^{(i)} = (\hat{I}_{\mathrm{x}}^{(i)}, \hat{I}_{\mathrm{y}}^{(i)}, \hat{I}_{\mathrm{z}}^{(i)})$ are the electronic and nuclear spin vectors, respectively, consisting of spin-1 matrices $\hat{S_\alpha}$ and spin-$\frac{1}{2}$ matrices $\hat{I}_{\alpha}^{(i)} = \hat{\sigma}_{\alpha}^{(i)}/2$ (with $\hat{\sigma}_{\alpha}^{(i)}$ the Pauli spin matrices). Furthermore, $\boldsymbol{A}^{(i)}$ is the electron-nuclear hyperfine tensor and $\boldsymbol{C}^{(ij)}$ is the nuclear-nuclear dipole-dipole coupling.

For the sensing schemes presented in this work, the NV-electron spin is either in the $m_s = +1$ or $m_s = -1$ eigenstate during evolution of the nuclear spins, except for sub-$\mu$s timescales between electron pulses. As a result of the disorder induced by the hyperfine interaction $|A_{\mathrm{zz}}{}^{(i)} - A_{\mathrm{zz}}{}^{(j)}| \gg C_{zz}{}^{(ij)}$, nuclear flip-flops are suppressed (frozen core). Note that this condition breaks down in general when there is a high degree of spectral crowding in the system. However, in this work, we focus on spectral regions where $C_{zz}{}^{(ij)}$ (denoted as $C_{ij}$ in the main text and from here on) is generally small if $|A_{\mathrm{zz}}{}^{(i)} - A_{\mathrm{zz}}{}^{(j)}|$ is small, so that the condition still holds. Furthermore, based on the large zero-field splitting $\Delta_{ZFS}$ we apply the secular approximation, so that the hyperfine tensor simplifies to just the parallel ($A_{\parallel} = A_{\mathrm{zz}}$) and perpendicular($A_{\perp} = \sqrt{A_{\mathrm{zx}}^2 + A_{zy}^2}$) components. Considering only the nuclear Hamiltonian while the electron is in the $\pm 1$ eigenstate:

$$\hat{H}_{\pm 1} = \sum_{i=1}^{N} \left[ (\gamma_{\mathrm{c}} B_{\mathrm{z}} \pm A_{\parallel}^{(i)}) \hat{I}_{\mathrm{z}}^{(i)} + A_{\perp}^{(i)} (\cos\phi_{\perp}^{(i)}\, \hat{I}_{\mathrm{x}}^{(i)} + \sin\phi_{\perp}^{(i)}\, \hat{I}_{\mathrm{y}}^{(i)}) \right] + \sum_{i=1}^{N}\sum_{j=i+1}^{N} \hat{\vec{I}}^{(i)} \cdot \boldsymbol{C}^{(ij)} \cdot \hat{\vec{I}}^{(j)}\,, \tag{2}$$

where $\phi_{\perp}^{(i)}$is the perpendicular hyperfine azimuthal angle. Here, we neglect the small correction on $C_{ij}$ due to the electron spin state ($C_{ij}^{+} = C_{ij}^{-}$) [1]. Supplementary Note 4 discusses the non-neglible effect of this correction observed in the specific experiments.

Under the application of a strong magnetic field ($\gamma_{\mathrm{c}} B_{\mathrm{z}} \pm A_{\parallel} \gg A_{\perp}$), we can further simplify the Hamiltonian, with the purpose of generalizing the effective dynamics of our system. To this end, we include the perpendicular hyperfine component ($A_{\perp}$) as a correction to the $\hat{I}_{\mathrm{z}}^{(i)}$ terms, describing the increased nuclear precession frequency [3]. This results in Eq. 1 in the main text:

$$\hat{H}_{\pm 1} \approx \sum_{i=1}^{N} A_i^{\pm} \hat{I}_{\mathrm{z}}^{(i)} + \sum_{i=1}^{N}\sum_{j=i+1}^{N} C_{ij} \hat{I}_{\mathrm{z}}^{(i)}\, \hat{I}_{\mathrm{z}}^{(j)}\,, \tag{3}$$

with $A_i^{\pm} = \sqrt{(\gamma_{\mathrm{c}} B_z \pm A_{\parallel}^{(i)})^2 + (A_{\perp}^{(i)})^2}$ the effective nuclear spin frequencies. In the main text, $A_i$ describes the spin frequencies when the electron is in the $m_s = -1$ state ($\pm$-sign is omitted for simplicity).

### 2. Initialisation

For the experiments in the main text, the nuclear spins are polarised via two techniques. We employ a combination of dynamical nuclear polarisation (PulsePol, [4]) and SWAP sequences with the electron spin [5]. The latter method typically yields a higher degree of polarisation [5], but is restricted to a limited number of spectrally isolated nuclear spins (see Supplementary Table I). If any of these SWAP-initialised spins participate in a sensing chain, we re-initialise them right before the final SEDOR-yx sequence to maximize the signal. All other spins are polarised via the PulsePol sequence, which is known to produce varying degrees of polarisation [2, 6, 7]. Following the definitions given in the supplement of Ref. [2], we set $\tau = 0.434\,\mu$s, resonant with the nuclear Larmor frequency, $N = 4$ and choose $R$ in the range $500 - 10000$, dependent on the rate of polarisation of the spins in the chain. For resonant reset of the electron state after each PulsePol step, the repump laser power was set to 1000 nW, with a repump time of 5 µs. For

some experiments (in particular the 2D spectroscopy) the laser power was reduced to 333 nW (and the repump time increased to 10 µs), to limit electron ionisation for $R > 5000$.

For the analytical expressions derived in the supplement, we capture the varying degree of polarisation of $N$ nuclear spins by a partially mixed system initial state:

$$\hat{\rho}_0 = \hat{\rho}_{e,0} \bigotimes_{i=1}^{N} \frac{1}{2} \left( \hat{\mathbb{I}} + p_i \hat{I}_z^{(i)} \right) , \tag{4}$$

with $\hat{\mathbb{I}}$ the (two-dimensional) identity matrix, the polarisation degree $p_i \in [-1, 1]$ for spin $i$ and $\hat{\rho}_{e,0}$ the initial state of the NV electron spin. The $p_i$ can be obtained by independent state preparation and measurement characterisations [2].

### *3. Readout*

For all experiments presented in this work, the signal is read out via dynamical-decoupling sensing sequences (DD or DDRF [3, 5]) with the electron spin. Only a limited number of spins can be read out selectively (see Supplementary Table I), due to spectral overlap between nuclear spins. Hence, we choose the first spin in all sensing chains to be one that is directly accessible to the electron spin. Combined initialisation and readout fidelity varies between spins ($0.44(2) - 0.95(2)$, corrected for electron readout fidelity).

## Supplementary Note 2: Spectral crowding in the NV-nuclear system

The schematic in the main text (Fig. 1), describes the challenge of mapping a spectrally crowded spin network in a general sense, for an abstract spin network described by frequencies $A_i, C_{ij}$ (Eq. 1). In the system studied here, all nuclear spins are $^{13}$C spins and the nuclear spin frequencies $A_i$ are set by the coupling to the NV electron spin. Therefore, there is a specific relationship between the nuclear-spin frequencies and their 3D position with respect to the electron spin (see also Supplementary Fig. 1). In this section we discuss how different spectral regions can be defined, and which regions can and cannot be accessed with different methods.

To understand which parts of the network can be mapped with the new methods, and where different methods break down, we define three spectral regions: *isolated*, *spectrally crowded* and *spatially crowded*. The *isolated* region is the set of spins $i$, for which:

$$|A_i - A_j| > 1/T_2^\star \ \forall \ j\,, \tag{5}$$

which states that the frequency of spin $i$ is unique. Here, we define this condition as being satisfied if there are at least 4 spectral widths (s.d.) between the resonances of two spins. In a natural abundance (1.1%) sample, we calculate that 20(3) spins typically satisfy this condition, and only about 8(3) are isolated by more than 2 kHz from any other spin (assuming $T_2^\star \sim 5$ ms and a purely dipolar hyperfine interaction). In the NV-nuclear system, only this set of spins can be read out selectively with the electron spin [1, 3, 5] (ignoring notable exceptions in the form of strongly-coupled spin-pairs [8, 9]), even though the electron spin typically couples to the majority of nearby nuclear spins ($\Delta_i > 1/T_{2,e}$).

Next, we define the *spectrally crowded* region as the set of spins $i$ for which:

$$|A_i - A_j| < 1/T_2^\star \implies C_{ij} \lesssim 1/T_2 \ \forall \ j\,, \tag{6}$$

meaning that spins may overlap spectrally, but if they do, they are typically not coupled strongly together. In the NV-nuclear system, this region includes nuclear spins which have similar hyperfine interaction with the electron, but are *spatially* separated, for example when they are on opposite sides of the NV center in the $x, y$-plane.

Finally, the *spatially crowded* region includes all other spins, which may be overlapping spectrally as well as coupling strongly together.

We schematically draw the three spectral regions in Supplementary Fig. 5, with the bottom color bar denoting the three spectral regions (green = *isolated*, orange = *spectrally crowded* and red = *spatially crowded*). The blue through violet bands indicate spin frequencies $A_i$ (analogous to the colored circles in the main text), shifted by the hyperfine interaction to the electron spin. In the schematic, we simplify the more complex dipolar isoplane shape [10] and consider only the radial dependence ($A_i \propto r_i^{-3}$). A particular nuclear-spin-network configuration is drawn as an example.

Only limited information can be attained from the network using pairwise SEDOR sequences [1]. If we allow no assumptions on the underlying coupling structure, pairwise measurements can only unambiguously assign couplings to the spins in the isolated region (coloured white). Using SEDOR, it is possible to measure couplings between one spin in the *isolated* region and one in the *spectrally crowded* region. However, those couplings cannot be assigned to a spin in the latter region without resorting to a detailed microscopic model [1]. As an example, using SEDOR, we find that the frequencies $A_1, A_2$ and $A_3$ all exhibit coupling to some spin at $A_4$ (coloured grey), but the couplings could belong to either of the spins at $A_4$. Also, couplings for which both spins lie in the *spectrally crowded* region (coloured black) cannot be accessed, as the spin-selective readout with the electron spin breaks down in this region.

### 1. *Spectral regions*

The spin-chain sensing (Fig. 2) unlocks new parts of the crowded region that can be mapped (Supplementary Fig. 5c). Consider the previously discussed couplings between $A_1, A_2, A_3$ and $A_4$. If we measure a chain connecting $A_1, A_4$ and $A_2$, we conclude that $A_1$ and $A_2$ couple to the same spin at $A_4$. Measuring a chain between $A_1, A_4$ and $A_2$ does not result in an observable coupling, so we conclude that $A_2$ is coupled to another spin at $A_4$. Note that this reasoning relies on the fact that it is possible to assert that $A_1, A_2$ and $A_3$ couple only to a *single* spin at $A_4$. Experimentally, this can be verified by observing a single dominant oscillation in the signal (instead of beatings or decay).

Besides assigning measured couplings to spins, spin-chain sensing allows for access to an extended number of couplings, particularly in the *spectrally crowded* and *spatially crowded* regions (Supplementary Fig. 5c). For example, by measuring a looped spin chain through frequencies $A_1, A_2, A_4, A_5, A_3, A_1$, we access the coupling between two spins in the spectrally crowded region (at $A_4$ and $A_5$) and directly map the connectivity of the 5 spins in the loop. In addition, we can use the newly unlocked spins (at $A_4$ and $A_5$) to probe couplings to the *spatially crowded* region

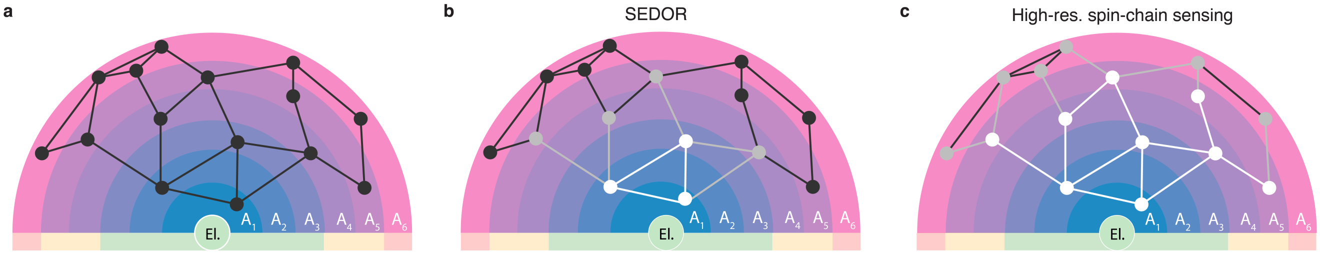


**Supplementary Fig. 5**. **Unlocking spectral regions. a** Alternative representation of Fig. 1, specific to the electron-nuclear system. Nuclear spins are drawn as black dots, connected by lines denoting observable couplings ($C_{ij} > 1/T_2$). The colored bands indicate spin frequencies $A_i$, shifted by the hyperfine interaction ($\Delta_i$) with the NV electron spin (mint green). The green, orange and red color scales at the bottom denote three spectral regions: *isolated*, *spectrally crowded* and *spatially crowded*, respectively. In the *isolated* region, spin frequencies are well-separated in frequency, (single spin per band) and can be read out directly with the electron [5]. In the *spectrally crowded* region (orange), multiple spins may occupy a single frequency band, but do not couple among each other. In the *spatially crowded* region (red), spins may also couple to spins within their frequency band, resulting in a decreased spin-echo coherence. **b** Spins that can be mapped using standard pairwise SEDOR [1]. The color of the coupling line indicates whether it can be measured and conclusively assigned (white), measured but not assigned (grey), or not measured at all (black). **c** Spin-chain sensing increases the number of couplings that can be measured and assigned (e.g. when *both* spins are in the *spectrally crowded* region) and unlocks spins in the *spatially crowded* region. Additionally, the high-resolution measurements of $\Delta_i$ allow for assigning otherwise ambiguous couplings in the *spectrally crowded* region.

(grey spins at $A_6$). Finally, by increasing the spectral resolution (Figs. 3 and 4), we resolve remaining ambiguity in the *spectrally crowded* region.

Even though the high-resolution spin-chain sensing fully unlocks the *spectrally crowded* region and allows us to probe the *spatially crowded* region, the latter also imposes a limit on the applicability of the method. In particular, the spin-chain sensing relies on an extended nuclear spin-echo coherence time (ideally from $T_2^\star$ to $T_2$). However, in this region, a decoupling pulse inadvertently also acts on other nuclei, so that their spin-spin couplings are retained. This results in the re-emergence of quasi-static ($T_2^\star$-like) noise also known as instantaneous diffusion [11], limiting the spin-echo coherence time $T_{2,\mathrm{SE}}$ to:

$$T_2^\star \leq T_{2,\mathrm{SE}} \leq T_2 \,. \tag{7}$$

In the case when many strongly coupled spins reside in the same frequency band (see Supplementary Eq. 6), we expect the coherence to be effectively reduced to $T_2^\star$, rendering the effect of the double resonance sequence useless. Hence, utilizing spins inside the *spatially crowded* region as probes of their environment is infeasible (except for strongly interacting spins, if $C_{ij} > 1/T_2^\star$), which sets the limit of the functional range of spin-chain sensing.

### 2. *Numerical simulations*

To quantitatively investigate the regions visualised in Supplementary Fig. 5, we perform Monte Carlo simulations of randomly generated NV-nuclear systems. First, we compute the spectral spin density (i.e. number of spins within a frequency bin) as a function of the hyperfine shift $\Delta$ (Supplementary Fig. 6a). We find that the mean spin density is well described by (dotted line):

$$\bar{\rho}(\Delta) = \frac{\pi^2\,\alpha\,\rho_0}{\Delta^2} \,, \tag{8}$$

with $\bar{\rho}$ the mean spin density in frequency space ($\mathrm{Hz}^{-1}$), $\rho_0 = 1.950\,\mathrm{nm}^{-3}$ the spatial $^{13}$C density and $\alpha = \mu_0\gamma_\mathrm{e}\gamma_\mathrm{c}\hbar/4\pi$, with $\mu_0$ the Bohr magneton, $\hbar$ the reduced Planck constant and $\gamma_\mathrm{e}$ and $\gamma_\mathrm{c}$ the electron and carbon nuclear gyromagnetic ratios, respectively. Supplementary Fig. 6a shows the expected number of $^{13}$C spins within a frequency bin of $100\,\mathrm{Hz} \sim 1/(2\,T_2^\star)$, which gives a measure for the probability to find spectrally overlapping spins. As described above, here we choose to define the start of the *spectrally crowded* region at the condition:

$$\bar{\rho} \approx T_2^\star \,, \tag{9}$$

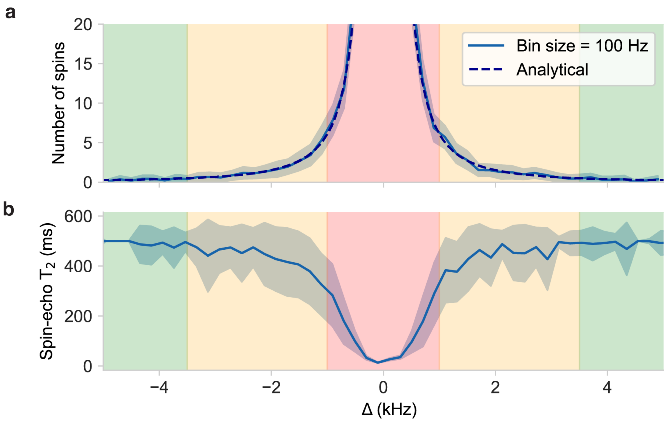


**Supplementary Fig. 6**. **Spectral spin-density and spin-echo coherence reduction.** **a** Expected spectral density (number of spins per 100 Hz) as a function of the hyperfine shift $\Delta$, assuming dipolar electron-nuclear coupling. The blue solid line denotes the mean of 100 randomly generated systems (containing $> 15000$ spins). The analytical expression in Supplementary Eq. 8 (dashed line) describes the simulated distribution well. **b** Expected spin echo coherence time (Eff. $T_2$) as a function of the hyperfine shift $\Delta$ (considering systems of $> 1000$ spins), taking into account only quasi-static effects and limiting the maximal coherence to $T_2 = 500$ ms. Coherence is reduced as the pulses in the spin-echo are resonant with multiple spins (see (a)), resulting in a recoupling of nearby spins. Here, the Rabi frequency is set to $f_{\mathrm{Rabi}} \approx 1/2T_2^\star$. Broader pulses (higher $f_{\mathrm{Rabi}}$) increase the number of recoupled spins, further reducing the nuclear coherence. Shaded areas denote the spread (one s.d.) between simulated systems .

yielding $|\Delta| \approx 3.5\,\mathrm{kHz}$. For larger $|\Delta|$, we expect on average less than one spin per $T_2^\star$-limited frequency bin (*isolated region*, see green region in Supplementary Fig 6a).

To quantify the transition between the *spectrally crowded* and *spatially crowded* regions, we compute the expected drop in spin-echo coherence, taking into account instantaneous diffusion [11] (Supplementary Fig 6b). Assuming linear addition of the dephasing rates [12]:

$$1/T_{2,\mathrm{SE}} = 1/T_2 + 1/T_{2,\mathrm{ID}}\,, \tag{10}$$

with $1/T_{2,\mathrm{ID}}$ the instantaneous diffusion dephasing rate and $T_2 \approx 500\,\mathrm{ms}$ the isolated spin-echo coherence time [5]. We compute $T_{2,\mathrm{ID}}$ by examining the mean $T_2^\star$ of the subsystem of spins that occupy a frequency bin of size 100 Hz for all $\Delta$. We mark the start of the *spatially crowded* region at the point $T_{2,\mathrm{SE}} \approx 0.5\,T_2$, which implies $|\Delta| \lesssim 1\,\mathrm{kHz}$ (Supplementary Fig. 6b). For the 23 newly characterised spins in this work, most belong to the *spectrally crowded* or *spatially crowded* regions (8 satisfy $3.5 < |\Delta_i| < 7.5$ kHz, 10 satisfy $1 < |\Delta_i| < 3.5$ kHz, and 4 satisfy $|\Delta_i| < 1$ kHz, see Supplementary Table I).

The discussion so far has focused on the degree of spectral crowding using the numerical values for a natural abundance sample (1.1% $^{13}$C). We now examine how these relations depend on the isotope concentration. In the basic picture of a point-dipole electron spin, surrounded by a bath of nuclear spins with spatial density $\rho_0$, the system exhibits a form of scale invariance. That is, we can define the (dimensionless) spectral density:

$$\frac{\bar{\rho}(\Delta)}{T_2^\star} = \frac{\pi^2\,\alpha\,\rho_0}{T_2^\star \Delta^2}\,, \tag{11}$$

describing the number of spins per line width. For dipolar interactions, both the electron-nuclear hyperfine shift $\Delta$ as well as the nuclear line width ($\sim 1/T_2^\star$) scale linearly with the density, so that the dimensionless spectral density (Supplementary Eq. 11) is independent of $\rho_0$. Intuitively, this can by understood as both the nuclear-spin line widths and the spacing between nuclear-spin frequencies scaling with $\rho_0$, keeping their ratio (i.e. the degree of spectral crowding) constant. In principle, in this elementary system, the physics and quantities like the number of spins that can be mapped are independent of concentration, with only absolute time and distances being rescaled.

In practice, however, this scale invariance breaks down for both for low and high isotope concentrations. At low isotope concentrations, other noise sources will start to limit both the nuclear line widths (via reduced $T_2^\star$), as well as the spin-echo coherence times (via reduced $T_2$). At high concentrations — including around the natural 1.1% abundance — the discreteness of the lattice and the contact hyperfine interaction due to the finite NV electron wave

function need to be taken into account. Predictions for the optimal concentration for a given goal, such as controlling the largest network, likely need detailed numerical simulations taking these details into account, which we do not pursue here.

## Supplementary Note 3: Spin-chain sensing

### 1. *Signal analysis*

Here, we analyse the system evolution under the spin-chain sensing sequences developed in this work, and give analytic expressions for the expected resulting signals. Given the initial state in Supplementary Eq. 4, we calculate the $z$-expectation value of the first nuclear spin (1) in the chain, for a chain of length $N$ after applying the concatenated SEDOR sequence (Fig. 2c). We analyse the evolution of the system by dividing it up into separate blocks, set by the subsequent SEDOR sequences. As the nuclear spins are initialized along the $z$-axis (have no off-diagonal component, see Supplementary Eq. 4), the spins in the chain do not evolve, except for those participating in a SEDOR block. Therefore, we can restrict our analysis to the subspace spanned by the spins in each block.

We find a recursive expression for the evolution of two subsequent spins ($j+1$ and $j$) under a SEDOR block (for both 'xx' and 'yx' type sequences). To this end, we trace out the $j+1$-subspace, as only the density matrix of spin $j$ is needed to calculate the subsequent SEDOR evolution between spin $j$ and $j-1$. This allows us to find two recursive formulas involving only the diagonal elements of each spin density matrix. By applying these expressions $N-1$ times, we retrieve the $z$-expectation value of spin 1, which is a function of all nuclear-nuclear couplings in the chain.

Evolution during a single SEDOR-xx or SEDOR-yx block for two subsequent spins in the chain can be described by the unitary:

$$U_{\mathrm{kx}}^{(j+1\to j)} = R_{\mathrm{x}}(\tfrac{\pi}{2})^{(j)}\ U_{-}\ R_{\mathrm{x}}(\pi)^{(j+1)}\ R_{\mathrm{x}}(\pi)^{(j)}\ U_{-}\ R_{\mathrm{k}}(\tfrac{\pi}{2})^{(j)}, \tag{12}$$

with $R_{\mathrm{k}}(\theta)^{(j)}$ a rotation of spin $j$ by an angle $\theta$ around axis $k \in \{x, y\}$ and $U_{-}$ the free evolution under the Hamiltonian $\hat{H}_{-1}$ in Supplementary Eq. 3, considering only spin $j+1$ and $j$ [2]. Without loss of generality, we can write the initial state of any two subsequent spins as:

$$\rho_{j+1,j}(0) = \frac{1}{4}\begin{pmatrix} 1+\alpha_{j+1} & \beta_{j+1} \\ \beta_{j+1}^{*} & 1-\alpha_{j+1} \end{pmatrix} \otimes \begin{pmatrix} 1+p_j & 0 \\ 0 & 1-p_j \end{pmatrix}, \tag{13}$$

where the first density matrix denotes the subspace of spin $j+1$, which can be in any arbitrary quantum state and the second density matrix describes the subspace of spin $j$, initialised according to Supplementary Eq. 4. We let the system evolve for time $t$ under $U_{\mathrm{kx}}^{(j+1\to j)}$, after which we trace out the $j+1$ subspace, resulting in the density matrix for spin $j$:

$$\begin{aligned} \rho_j(t) &= \mathrm{Tr}_{j+1}\left( U_{\mathrm{kx}}^{(j+1\to j)}\ \rho_{j+1,j}(0)\ U_{\mathrm{kx}}^{(j+1\to j)\dagger} \right) \\ &= \frac{1}{2}\begin{pmatrix} 1+\alpha_j^k & \beta_j^k \\ {\beta_j^k}^{*} & 1-\alpha_j^k \end{pmatrix}, \end{aligned}$$

leading to the following update rule for the diagonal density matrix elements of spin $j$ under SEDOR-xx and SEDOR-yx:

$$\alpha_j^x = p_j \cos\frac{2\pi C t}{2}, \tag{14}$$

$$\alpha_j^y = p_j \sin\frac{2\pi C t}{2}\,\alpha_{j+1}, \tag{15}$$

with $C = C_{j,j+1}$ the coupling between the spins in Hz. Note that the off-diagonal terms $\beta_{j+1}$ drop out when we only consider the diagonal elements of spin $j$. To calculate the $z$-expectation value of the first spin in the chain after a SEDOR-xx block and $N-2$ concatenated SEDOR-yx blocks according to Fig. 2c, we iteratively apply Supplementary Eqs. 14 and 15 to find:

$$\left\langle \hat{I}_{\mathrm{z}}^{(1)} \right\rangle = \frac{1}{2}\alpha_1 = \frac{1}{2} p_{N-1} \cos\left(\pi C_{N-1,N} t_{N-1,N}\right) \prod_{j=1}^{N-2} p_j \sin\left(\pi C_{j,j+1} t_{j,j+1}\right), \tag{16}$$

*2. Decoherence of the chain*

Supplementary Eq. 16 does not take into account any imperfections due to decoherence or pulse errors. In the following, we model the effect of decoherence, which is the main factor limiting the signal. Here, we do not take into account the effect of pulse errors, but this can be implemented analogous to Ref. [2].

We model decoherence by multiplying the signal of each SEDOR block by an exponential decay function, parameterised by a characteristic spin-echo decay time $\tau_j$. In the case that the spin-echo in the SEDOR is perfectly effective, meaning that spin $j$ is fully decoupled from all other nuclei, the decay is governed by dynamic noise sources ($\tau_j \sim 250 - 800$ ms) [5]. However, for a spin at a spectrally crowded frequency, the decoupling pulse inadvertently also acts on other nuclei, so that their coupling to spin $j$ is retained. This results in the re-emergence of quasi-static noise (instantaneous diffusion) discussed in Supplementary Note 2, which we model by adding a Gaussian decay to Supplementary Eq. 16:

$$\left\langle \hat{I}_{\mathrm{z}}^{(1)} \right\rangle = \frac{1}{2} p_{N-1} \cos\left(\pi C_{N-1,N} t_{N-1,N}\right) e^{-\left(\frac{t_{N-1,N}}{\tau_{N-1}}\right)^2} \prod_{j=1}^{N-2} p_j \sin\left(\pi C_{j,j+1} t_{j,j+1}\right) e^{-\left(\frac{t_{j+1,j}}{\tau_j}\right)^2}, \tag{17}$$

Even though the recoupled spins are partially polarised, we expect them to impart only a decay and no frequency shift on the signal, as they will also undergo the $\frac{\pi}{2}$-pulse, negating any $z$-axis polarisation. For the experiments in Fig. 2d-f, we set $t_{i,i+1} = \frac{1}{2} C_{i,i+1}^{-1}$ for all SEDOR-yx blocks, which reduces Supplementary Eq. 17 to:

$$\left\langle \hat{I}_{\mathrm{z}}^{(1)} \right\rangle = A_{N-1} \cos\left(\pi C_{N-1,N} t_{N-1,N}\right) e^{-\left(\frac{t_{N-1,N}}{\tau_{N-1}}\right)^2}, \tag{18}$$

with $A_{N-1} = \frac{1}{2} p_{N-1} \prod_{j=1}^{N-2} p_j e^{-\left(\frac{C_{j+1,j}}{2\tau_j}\right)^2}$ the signal amplitude. We use Supplementary Eq. 18 to fit the data in Fig. 2d-f with free parameters $A_{N-1}, C_{N-1,N}, \tau_{N-1}$ and an arbitrary offset. Note that even though the signal amplitude $A_{N-1}$ is affected by the coherence and polarisation of all spins in the chain, the spectral resolution with which $C_{N-1,N}$ can be determined is only limited by the coherence of spin $N-1$ (i.e. $\tau_{N-1}$). The decay of the signal $A_{N-1}$ with increasing number of spins due to the imperfect polarisation ($p_j < 1$) and finite coherence times $\tau_j$ limits how long a chain can be practically formed. Note that for spins further from the NV $\tau_j$ tends to decrease due to imperfect decoupling (instantaneous diffusion in the spatially crowded region, see Supplementary Note 2), ultimately limiting the range for high-resolution sensing.

## Supplementary Note 4: Electron-nuclear double resonance sequence

### 1. Signal analysis

Next, we analyse the system evolution under the electron-nuclear double resonance sequence (see Fig. 3). To this end, we consider the interaction between the electron ('el') and nuclear spin $j$ (Fig. 3) by considering the following unitary:

$$U_{\mathrm{xx}}^{(\mathrm{el}\to j)} = R_{\mathrm{x}}(\tfrac{\pi}{2})^{(j)}\; U_{+}\; R_{\mathrm{x}}(\pi)^{(j)}\; U_{-}\; R_{\mathrm{x}}(\tfrac{\pi}{2})^{(j)}\,, \tag{19}$$

with $U_{\pm}$ the unitary evolution under Hamiltonian $\hat{H}_{\pm 1}$ (Supplementary Eq. 3). We compute the $z$-expectation value of nuclear spin $j$ after applying $U_{\mathrm{xx}}^{(\mathrm{el}\to j)}$ (starting in initial state given by Supplementary Eq. 4):

$$\left\langle \hat{I}_{\mathrm{z}}^{(j)} \right\rangle = \frac{1}{2} p_j \cos\left(2\pi\Delta_j\, t\right). \tag{20}$$

Note that for some experiments in this work, the final $\pi/2$-rotation was performed along the $-x$-axis instead of the $x$-axis, which leads to a minus sign on the signal, but has no impact on the frequency or amplitude. The frequency (in Hz) is defined as the hyperfine shift referred to in the main text:

$$\Delta_j = \frac{1}{2}(A_j^{-} - A_j^{+})\,, \tag{21}$$

$\Delta_j$ provides a high-resolution measurement of the frequency shift due to the electron-nuclear hyperfine interaction. In this work, the function of this measurement is to distinguish different spins with similar precession frequencies. That is, $\Delta_j$ provides a high-resolution label for the spins.

An additional application is to perform precise spectroscopy of the system to determine the hyperfine interaction, for example for comparison to density functional theory calculations [13] or to determine the Hamiltonian parameters for developing precise quantum control. Next, we analyze the relation of $\Delta_j$ to the hyperfine parameters.

We use the spin frequencies $A_j^{\pm}$ introduced in Supplementary Eq. 3, but now allow for a slight misalignment of the external magnetic field away from the NV-axis (z-axis):

$$\Delta = \frac{1}{2}\left(\sqrt{(\gamma_{\mathrm{c}}B_{\mathrm{z}} - A_{\mathrm{zz}})^2 + (\gamma_{\mathrm{c}}B_x - A_{\mathrm{zx}})^2 + (\gamma_{\mathrm{c}}B_y - A_{zy})^2} - \sqrt{(\gamma_{\mathrm{c}}B_{\mathrm{z}} + A_{\mathrm{zz}})^2 + (\gamma_{\mathrm{c}}B_x + A_{\mathrm{zx}})^2 + (\gamma_{\mathrm{c}}B_y + A_{zy})^2}\right), \tag{22}$$

where we omit the spin-subscript $j$ for readability. Here, $B_x$ and $B_y$ are the perpendicular field components. Note that for simplicity we use a purely geometric argument and do not take into account spin mixing for the eigenstates (i.e. the eigenstates are set as the electron and nuclear spin states), which would introduce additional (small) frequency shifts. Supplementary Eq. 22 shows that measuring $\Delta$ for different magnetic field vectors makes it possible to determine the hyperfine parameters, given that the field components are known. As the magnetic field components generally are not exactly known, we now analyze various situations and approximations.

For a strong field aligned along the z-axis, the perpendicular hyperfine components are a small perturbation:

$$\Delta = \frac{1}{2}\left(\sqrt{(\gamma_{\mathrm{c}}B_{\mathrm{z}} - A_{\mathrm{zz}})^2 + {A_{\perp}}^2} - \sqrt{(\gamma_{\mathrm{c}}B_{\mathrm{z}} + A_{\mathrm{zz}})^2 + {A_{\perp}}^2}\right) \tag{23}$$

$$\approx -A_{\mathrm{zz}}\left(1 - \frac{{A_{\perp}}^2}{2(\gamma_{\mathrm{c}}B_{\mathrm{z}} - A_{\mathrm{zz}})(\gamma_{\mathrm{c}}B_{\mathrm{z}} + A_{\mathrm{zz}})}\right). \tag{24}$$

Supplementary Eq. 23 shows that the measurement predominantly probes the hyperfine component parallel to the magnetic field. For the magnetic field used in this work ($B_{\mathrm{z}} \sim 403$ G) and typical hyperfine values ($A_{\mathrm{zz}} \sim A_{\perp} \sim 10$ kHz), the typical deviation from $A_{\mathrm{zz}}$, due to $A_{\perp}$, is smaller than 0.03 % (less than 3 Hz). Note that the effect of a finite $A_{\perp}$ is suppressed because both terms in the top line of Supplementary Eq. 23 tend to shift in the same manner.

A misaligned field, with non-zero $B_x$ and $B_y$ components, in combination with a non-zero $A_{\perp}$, causes an additional frequency shift of $\Delta$. Due to the sign difference in the first and second terms in Supplementary Eq. 22, the effect is relatively large and a perpendicular field of $\sim 0.5$ G causes a frequency shifts of a few Hz.

These results show that additional measurements and/or analysis are required to fully exploit the high-spectral-resolution measurements presented here for precision spectroscopy of the Hamiltonian parameters. However, this does not affect the capability used in this work to resolve different spins with high spectral resolution.

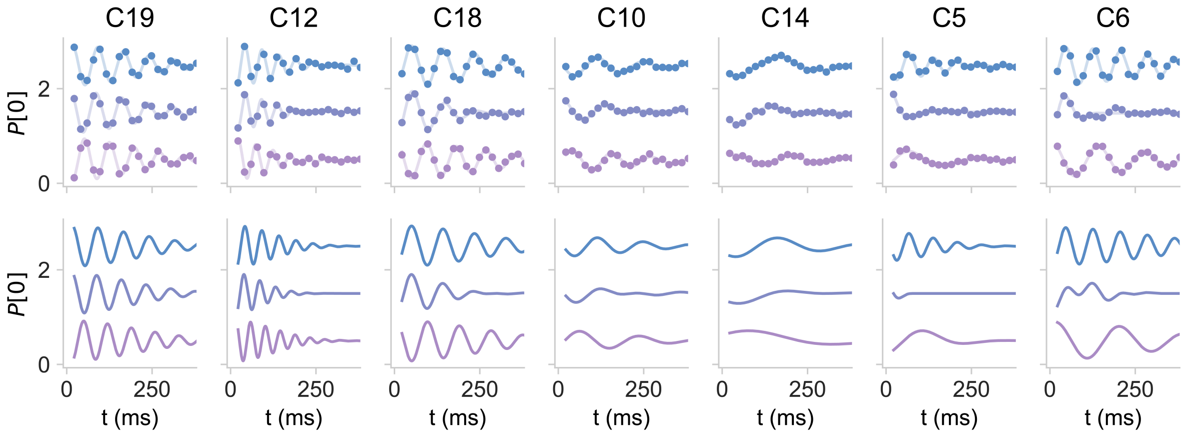


**Supplementary Fig. 7**. **Simulating electron-state-dependent dephasing for various nuclear spins** Top: Experimental data (as in Supplementary Fig. 4) for seven different nuclear spins after initialising the spin bath 'up' (blue), 'down' (pink) or in a 'mixed' (purple) state (offset for clarity). The 'mixed' signals exhibit a highly spin-dependent coherence decay, while polarising the bath leads to increased coherence and a shift in the frequency. The experimental data are corrected for difference in polarisation direction for selective and global initialisation (which leads to a minus sign). Bottom: Analytical model for the signal of the spins, obtained by evaluating Supplementary Eqs. 31 and 30, reproducing both the observed coherence decay and frequency shifts qualitatively, taking into account the couplings to all other 49 spins. The amplitude, phase, frequency and (limiting) $T_2$-decay of the base signal are extracted from a fit to the 'up' data.

### 2. *Pulse errors*

To investigate how pulse errors affect the measured frequency $\Delta$, we model the first $\pi/2$-pulse and the spin-echo $\pi$-pulse on nuclear spin $j$ as imperfect X-rotations with excitation probability $f^2$:

$$R_{\mathrm{x}}(f)^{(j)} = \sqrt{1-f^2}\,\hat{\mathbb{I}} - if\hat{I}_{\mathrm{x}}^{(j)}\,, \tag{25}$$

leading to the adapted unitary (Supplementary Eq. 19):

$$U_{\mathrm{xx}}^{(\mathrm{el}\to j)} = R_{\mathrm{x}}(\tfrac{\pi}{2})^{(j)}\;U_+\;R_{\mathrm{x}}(f_2)^{(j)}\;U_-\;R_{\mathrm{x}}(f_1)^{(j)}\,. \tag{26}$$

We find the signal contains three frequency components:

$$\left\langle \hat{I}_{\mathrm{z}}^{(j)} \right\rangle = \kappa_1 \cos\left(\Delta_j\, t\right) + \kappa_2 \cos\left(\omega_{\mathrm{L}}\, t\right) + \kappa_3 \cos\left(\frac{1}{2} A_j^{+}\, t\right), \tag{27}$$

with $\kappa_1$, $\kappa_2$, $\kappa_3$ some real constants, determined by the pulse excitation probabilities $f_1^2$ and $f_2^2$. For perfect $\pi/2$-pulse and $\pi$-pulse, only the first term remains, corresponding to the hyperfine shift that we aim to measure. The second term arises from the spin-echo $\pi$-pulse not being effective, so that the hyperfine interaction with the electron spin cancels. The third term arises from the $\pi/2$-pulse not exciting the nuclear spin and the $\pi$-pulse creating some coherence, analogous to performing a Ramsey during the second half of the sequence (when the electron is in the $m_s = +1$ state). These spurious frequencies are easily identifiable in the signal, as $\omega_{\mathrm{L}}$ and $A_j^{+}$ are typically $> 100$ kHz, while $\Delta_j$ ($\sim 1 - 50$ kHz) is tightly bound by the bandwidth of the RF pulses ($\sim 1$ kHz). Furthermore, the second and third terms decay quickly ($T_2^{\star}$-limited), as the spin-echo is not effective. Hence, any signal remaining after $\sim 10$ ms contains only the $\Delta_j$ term of interest.

### 3. *Electron-state-dependent dephasing*

At finite $B_{\mathrm{z}}$ field, a particular dephasing mechanism that we denote electron-state-dependent dephasing (ESD), prevents nuclear spins from attaining the full $T_2$ coherence time. The magnitude of this effect is highly spin-dependent and can be accurately modelled using the nuclear-spin interactions obtained here. As detailed in Ref. [1], the spin-spin

couplings ($C_{ij} \neq C^{+}_{ij} \neq C^{-}_{ij}$), weakly depend on the electron spin state, resulting in an effective frequency shift of all spin-spin couplings during the electron-nuclear double resonance sequence (Supplementary Eq. 19):

$$\Delta C_{ij} = \frac{1}{2}\left(C^{+}_{ij} - C^{-}_{ij}\right) . \tag{28}$$

The dominant contribution is due to a change in the nuclear quantisation axes [1]:

$$\Delta C_{ij} \approx \frac{(A^{(i)}_{\mathrm{zx}} + A^{(j)}_{\mathrm{zx}})C^{(ij)}_{zx} + (A^{(i)}_{zy} + A^{(j)}_{zy})C^{(ij)}_{zy}}{\gamma_{\mathrm{c}} B_{\mathrm{z}}} , \tag{29}$$

with $A^{(i)}_{z\alpha}$ and $C^{(ij)}_{z\alpha}$ the perpendicular components of the hyperfine and nuclear-nuclear dipole tensor, respectively. The key insight is that the couplings of spin $j$ to the network change between the first and second half of the spin-echo sequence. With the surrounding spin bath in a mostly mixed state (Supplementary Eq. 4), the quasi-static noise is not completely eliminated by the spin echo, as the $\Delta C_{ij}$-terms do not cancel. Typically these terms do not exceed $\sim 2$ Hz (see also Ref. [1]), but for some spins with strong couplings to the electron spin ($A_{\perp} \gg 10$ kHz), the effect leads to a loss of coherence comparable to $T_2^{\star}$ decay (see Supplementary Fig. 7, C5 and C6).

To model the effect on the measured signal, we consider two limiting cases: one where the surrounding spin bath is in a completely polarised state (denoted by ‘up’ or ‘down’ in Supplementary Fig. 7) and one in which it is in a mixed state. Considering the effect of $N$ nuclear spins on a target nuclear spin $j$, we find that the signal frequency gets shifted when the bath is polarised ($p_i = \pm 1 \,\forall\, i$ in Supplementary Eq. 4).

$$\left\langle \hat{I}^{(j)}_{\mathrm{z}} \right\rangle = \frac{1}{2} p_j \cos\left[2\pi(\Delta_j \pm \phi_j)\, t\right] , \quad \phi_j = \sum_{i}^{N} \frac{\Delta C_{ij}}{2} , \tag{30}$$

where the $\pm$-sign is given by the direction of the bath polarisation (‘up’ or ‘down’).

Next, assuming a mixed state for the bath spins ($p_i = 0 \,\forall\, i$ in Supplementary Eq. 4), we find:

$$\left\langle \hat{I}^{(j)}_{\mathrm{z}} \right\rangle = \frac{1}{2} p_j \cos\left(2\pi\Delta_j\, t\right) \prod_{i=1}^{N} \cos\left(2\pi\, \frac{\Delta C_{ij}}{2}\, t\right) \tag{31}$$

In this case, the coupled spins will cause frequency beatings on the signal, leading to a decay:

$$\left\langle \hat{I}^{(j)}_{\mathrm{z}} \right\rangle \approx \frac{1}{2} p_j \cos\left(2\pi\Delta_j\, t\right) e^{-(t/T_{2,\mathrm{ESD}})^2} \tag{32}$$

with characteristic decay time:

$$T_{2,\mathrm{ESD}} = \sqrt{\frac{2}{\sum_{i=1}^{N}\left(2\pi\, \frac{\Delta C_{ij}}{2}\right)^2}} \tag{33}$$

Supplementary Fig. 7b-e shows the experimental observation of the ESD effect for seven selected spins in both polarised and mixed spin bath conditions. These conditions are achieved by using either the global PulsePol sequence or a selective SWAP initialisation of the target nuclear spin (Methods). The obtained signals display significant coherence and frequency variation between spins and depend strongly on the state of the bath.

To model this behaviour, we first extract the frequency, amplitude, phase and coherence time of the ‘up’ data. Next, we calculate how the signal should change for different bath states due to the ESD effect, using Supplementary Eqs. 31 and 30 to generate a spin-specific model. To this end, the $\Delta C_{ij}$ for each of the spins are calculated according to Supplementary Eq. 29, based solely on the known spin positions (assuming dipolar hyperfine coupling). Supplementary Fig. 7f-i shows the modelled signal, for which we observe good qualitative agreement for each of the spins. The difference in initial phase between ‘up’ and ‘mixed’ data for some spins (Supplementary Fig. 7b,e) is due to a difference in polarisation direction between the selective and global initialisation sequences. Furthermore, using experimentally determined values of $\Delta C_{ij}$ [1] (since we know Supplementary Eq.29 to be approximate), further diminishes the discrepancy between model and data.

Typically, spins that are more strongly coupled to the electron spin (e.g. panel b) show a quick decoherence behaviour for unpolarized environments(see Supplementary Eq. 29). For more weakly coupled spins, the decoherence becomes determined by the basic $T_2$-echo time. For such more weakly coupled spins as well for highly polarised baths, we expect multiple refocussing pulses can further enhance coherence and therefore the resolution of the sequence.

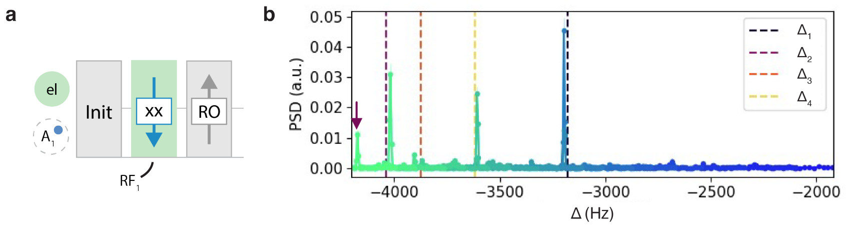


**Supplementary Fig. 8**. **Direct nuclear spectroscopy.** **a** Experimental sequence for performing double resonance spectroscopy directly with the electronic quantum sensor (as in Fig. 3). The pulses and readout can be tuned so that only signal from spins in a frequency region of interest ($A$) is picked up. This region of interest is then swept over a larger region to create a stitched spectrum. **b** Stitched spectrum (color shading shows individual datasets) as a function of hyperfine shift $\Delta$, demonstrating transform-limited spectral resolution ($\sim$ 10 Hz). Dashed lines denote the estimated $\Delta_i$ of four previously characterized spins in the frequency region. The purple arrow denotes a spurious alias, corresponding to $\Delta_1$. The error is smaller than the data points (calculated according to Ref. [14]).

### 4. *Electron-nuclear double resonance spectroscopy*

Next, we discuss how to use the electron-nuclear double resonance sequence to perform *direct* high-resolution spectroscopy of nuclear spins. Since the sequence only enhances coherence for spins resonant with the RF-pulses, we take multiple data sets at varying RF frequencies and stitch them together to create a larger scan. To this end, we implement the sequence sketched in Supplementary Fig. 8a. For the spectroscopy data in Supplementary Fig. 8b, we sweep frequency $\mathrm{RF}_1 \approx \omega_\mathrm{L} - \Delta$ from $\sim 434 - 436$ kHz ($\mathrm{RF}_1$ in the 'xx'-block), keeping the pulse Rabi frequency at 0.38 kHz and updating the DD readout parameters as ('RO'-block in Supplementary Fig. 8):

$$\tau_\mathrm{DD} = \frac{k}{4\,(\omega_\mathrm{L} - \Delta/2)}\,, \tag{34}$$

with $k = 41$ the DD resonance order and keeping the number of pulses fixed to $N = 208$. The update rule ensures that the readout remains resonant with the frequency of interest ($A_1$) [3, 15]. For each frequency, we sweep the double resonance evolution time $t$ (Supplementary Fig. 8a) up to 200 ms (bandwidth of 200 Hz) and compute the PSD, which we stitch together to create the large bandwidth, high resolution spectroscopy data (Supplementary Fig. 8b).

The spectral region interrogated in Supplementary Fig. 8b is known to contain four nuclear spins (dotted lines), three of them being visible in the signal with almost transform-limited linewidth ($\sim$ 10 Hz). The amplitude of the signal is determined by a combination of polarisation efficiency (see Supplementary Eq. 20) and readout fidelity [3, 8]. The colored arrow denotes suspected aliasing, which can be easily mitigated by increasing the sampling bandwidth.

The double resonance spectroscopy presented here can be readily implemented for other color center platforms [16–19] to interrogate the nuclear spin environment with high spectral resolution. By using DDrf readout sequences, the protocol is further simplified and can be used for nuclear spins with small perpendicular hyperfine coupling [5].

### 5. *Electron-nuclear double resonance of the 50-spin-network*

We perform the electron-nuclear double resonance sequence on all known spins in the network. As we can only access a number of spins directly, we implement the electron-nuclear double resonance block with a spin chain of varying length (See Supplementary Fig. 9). The used sequence for each spins can be found in Supplementary Table I.

To retrieve the absolute signal frequency from undersampled data, we take at least two data sets with different bandwidths and sampling rates [20]. Next, we correct for aliasing by minimizing the mean squared error between the multiple measurements, and selecting the most likely alias. We use prior knowledge of the $m_s = -1$ frequency of the spin to limit this analysis to a frequency range of 400 Hz around the expected resonance.

The values obtained for $\Delta_i$ are shown in Supplementary Table I, where the error denotes the weighted error on the mean of multiple measurements (whose error is determined from an exponentially decaying fit). For many of the previously characterised spins [1], we find good agreement with Ramsey measurements. However, for some spins (most notably C19), we find a deviation that cannot be explained by off-axis fields or a correction due to the (reported) Fermi contact term [13]. Further research is needed to identify the discrepancy between the Ramsey method used in Ref. [1] and values reported in this work.

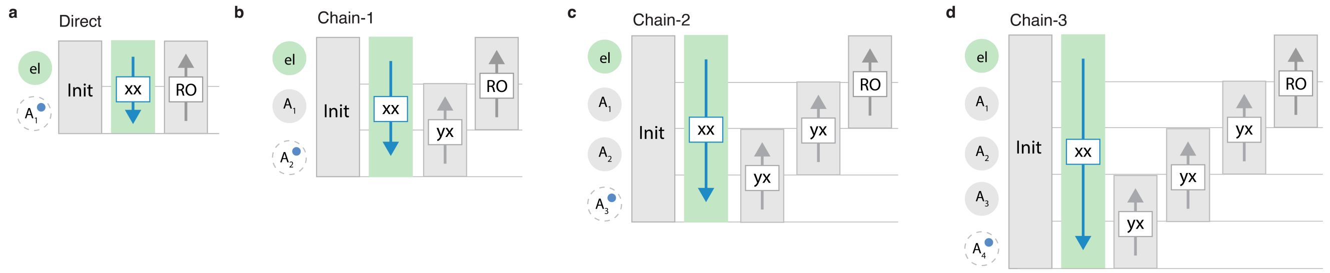


**Supplementary Fig. 9**. **Electron-nuclear double resonance of the spin-network**. **a-d** Pulse sequences used in this work to extract $\Delta_i$ for all spins in Supplementary Table I. We try to use chains of minimum length to limit experimental time and complexity. The exact sequence that was used is denoted in the 'Readout' column in Supplementary Table I.

## Supplementary Note 5: Network reconstruction algorithm

The network mapping procedure outlined in the Methods section relies on the (pseudo) function CheckVertex($w, T$). Here, we present pseudo-code for the procedure of checking whether vertex $w$ has already been mapped in $T$.

```
function CheckVertex(w, T)          ▷ Checks if w was already characterised and returns the duplicate vertex

    unique = True                   ▷ Boolean, keeps track whether vertex w is unique
    duplicate = None                ▷ Specifies duplicate vertex if applicable, otherwise None

    for k in T do
        if |A_w − A_k| < σ_A then   ▷ Compare frequency of w to mapped vertices, σ_A denotes uncertainty
            unique = False          ▷ w and k might be duplicates

            V_0^w = {w}             ▷ Create a spanning tree T_w with root w
            i = 0                   ▷ Keeps track of depth of search
            j = 0                   ▷ Keeps track of number of equivalent edges
            while not unique and duplicate == None and i < maxdepth do
                for each vertex v^w ∈ V_i^w and v^k ∈ V_i^k do   ▷ Find equivalent vertex in spanning tree T_k with root k
                    Δ = MeasureDelta(v^w)            ▷ Electron-nuclear double resonance measurement
                    if |Δ − Δ_k| > σ_Δ then          ▷ σ_Δ denotes uncertainty
                        unique = True                ▷ w is unique, different Δ
                    end if
                    if |Δ − Δ_k| < σ_Δ and σ_Δ < threshold then
                        unique = False               ▷ w is equal to k, similar Δ unlikely
                        duplicate = k
                    end if
                    for each vertex r^k ∈ V_{i+1}^k do   ▷ Get known neighbours of k from T_k
                        C = MeasureCoupling(v^w, A_r^k)  ▷ Between vertex v^w in T_w and the frequency of r^k in T_k
                        if |C − C_{vr}^k| > σ_C then     ▷ Compare to edge in T_k
                            unique = True                ▷ w is unique, different spanning tree
                        end if
                        if |C − C_{vr}^k| < σ_C and σ_C < threshold then   ▷ Measurement has reasonable uncertainty
                            j = j + 1                    ▷ Another edge of the spanning tree coincides
                            create r^w in T_w            ▷ Expand T_w
                            A_r^w = A_r^k
                            C_{vr}^w = C
                            add r^w to V_{i+1}^w in T_w
                        end if
                    end for
                end for
                i = i + 1
            end while
            if j > equaledges then
                unique = False
                duplicate = k               ▷ w and k are the same vertex, similar spanning tree unlikely
            end if
        end if
    end for

    return unique, duplicate
end function
```

The function starts by comparing the ($T_2^\star$-limited) frequency $A_w$ of vertex $w$ to the frequencies of all mapped vertices in $T$. If the frequencies coincide within the measurement uncertainty ($\sigma_A \sim 100$ Hz) for a vertex $k$, we initiate a procedure to check whether $w$ is that vertex. We do this by comparing information we have on vertex $k$ and its surroundings to specific measurements taken from $w$. In particular, we measure the spanning tree $T_w$ with

root $w$, and compare it to the spanning tree $T_k$ with root $k$ (for some maximum depth). If all couplings ($C_{ij}$, up to some threshold 'equaledges') and hyperfine frequency shifts ($\Delta_i$) of the two spanning trees are the same, we conclude $w$ and $k$ are the same vertex. If we measure a single deviation, we conclude $w$ must be unique. If the procedure is inconclusive, for example because the uncertainty of all measurements is large, 'duplicate' remains `None`.

---


[1] Abobeih, M. H. *et al.* Atomic-scale imaging of a 27-nuclear-spin cluster using a quantum sensor. *Nature* **576**, 411–415 (2019).

[2] Randall, J. *et al.* Many-body–localized discrete time crystal with a programmable spin-based quantum simulator. *Science* **374**, 1474–1478 (2021).

[3] Taminiau, T. H. *et al.* Detection and Control of Individual Nuclear Spins Using a Weakly Coupled Electron Spin. *Phys. Rev. Lett.* **109**, 137602 (2012).

[4] Schwartz, I. *et al.* Robust optical polarization of nuclear spin baths using Hamiltonian engineering of nitrogen-vacancy center quantum dynamics. *Sci. Adv.* **4**, eaat8978 (2018).

[5] Bradley, C. E. *et al.* A Ten-Qubit Solid-State Spin Register with Quantum Memory up to One Minute. *Phys. Rev. X* **9**, 031045 (2019).

[6] Rao, D. D. B., Ghosh, A., Gelbwaser-Klimovsky, D., Bar-Gill, N. & Kurizki, G. Spin-bath polarization via disentanglement. *New J. Phys.* **22**, 083035 (2020).

[7] Villazon, T., Chandran, A. & Claeys, P. W. Integrability and dark states in an anisotropic central spin model. *Phys. Rev. Research* **2**, 032052 (2020).

[8] Abobeih, M. H. *et al.* One-second coherence for a single electron spin coupled to a multi-qubit nuclear-spin environment. *Nat Commun* **9**, 2552 (2018).

[9] Bartling, H. P. *et al.* Entanglement of Spin-Pair Qubits with Intrinsic Dephasing Times Exceeding a Minute. *Phys. Rev. X* **12**, 011048 (2022).

[10] Perunicic, V. S., Hill, C. D., Hall, L. T. & Hollenberg, L. A quantum spin-probe molecular microscope. *Nat Commun* **7**, 12667 (2016).

[11] Tyryshkin, A. M. *et al.* Electron spin coherence exceeding seconds in high-purity silicon. *Nature Mater* **11**, 143–147 (2012).

[12] Bauch, E. *et al.* Ultralong Dephasing Times in Solid-State Spin Ensembles via Quantum Control. *Phys. Rev. X* **8**, 031025 (2018).

[13] Nizovtsev, A. P. *et al.* Non-flipping $^{13}$ C spins near an NV center in diamond: Hyperfine and spatial characteristics by density functional theory simulation of the C $_{510}$ [NV]H $_{252}$ cluster. *New J. Phys.* **20**, 023022 (2018).

[14] Hoyng, P. An error analysis of power spectra. *Astron. & AstroPhys.* **47**, 449–452 (1976).

[15] Degen, C. L., Reinhard, F. & Cappellaro, P. Quantum sensing. *Rev. Mod. Phys.* **89**, 035002 (2017).

[16] Debroux, R. *et al.* Quantum Control of the Tin-Vacancy Spin Qubit in Diamond. *Phys. Rev. X* **11**, 041041 (2021).

[17] Sipahigil, A. *et al.* An integrated diamond nanophotonics platform for quantum-optical networks. *Science* **354**, 847–850 (2016).

[18] Bourassa, A. *et al.* Entanglement and control of single nuclear spins in isotopically engineered silicon carbide. *Nature Materials* **19**, 1319–1325 (2020).

[19] Durand, A. *et al.* Broad Diversity of Near-Infrared Single-Photon Emitters in Silicon. *Phys. Rev. Lett.* **126**, 083602 (2021).

[20] Boss, J. M., Cujia, K. S., Zopes, J. & Degen, C. L. Quantum sensing with arbitrary frequency resolution. *Science* **356**, 837–840 (2017).
